# Expression-analysis of the human endogenous retrovirus HERV-K in human astrocytic tumors

**DOI:** 10.1186/1756-0500-7-159

**Published:** 2014-03-19

**Authors:** Almuth Friederike Kessler, Miriam Wiesner, Joachim Denner, Ulrike Kämmerer, Giles Hamilton Vince, Thomas Linsenmann, Mario Löhr, Ralf-Ingo Ernestus, Carsten Hagemann

**Affiliations:** 1 Tumorbiology Laboratory, Department of Neurosurgery, University of Würzburg, Josef-Schneider-Str. 11, Würzburg D-97080, Germany; 2 Robert Koch Institute, Nordufer 20, Berlin D-13353, Germany; 3 Department of Obstetrics and Gynaecology, University of Würzburg, Josef-Schneider-Str. 4, Würzburg D-97080, Germany; 4 Current address: Klinikum Klagenfurt am Wörthersee, Department of Neurosurgery, Feschnigstr. 11, Klagenfurt 9020, Austria

**Keywords:** Human endogenous retrovirus, HERV-K, Glioblastoma multiforme, Astrocytic tumor, Expression, Glioblastoma cell line, PCR analysis

## Abstract

**Background:**

The human endogenous retrovirus K (HERV-K) has been acquired by the genome of human ancestors million years ago. It is the most complete of the HERVs with transcriptionally active *gag*, *pol* and *env* genes. Splice variants of *env*, which are *rec*, 1.5 kb transcript and *Np9* have been suggested to be tumorigenic. Transcripts of HERV-K have been detected in a multitude of human cancers. However, no such reports are available concerning glioblastomas (GBM), the most common malignant brain tumor in adults. Patients have a limited prognosis of 14.6 months in median, despite standard treatment. Therefore, we elucidated whether HERV-K transcripts could be detected in these tumors and serve as new molecular target for treatment.

**Findings:**

We analyzed human GBM cell lines, tissue samples from patients and primary cell cultures of different passages for HERV-K full length mRNA and *env*, *rec* and 1.5 kb transcripts. While the GBM cell lines U138, U251, U343 and GaMG displayed weak and U87 strong expression of the full length HERV-K, the splice products could not be detected, despite a weak expression of *env* mRNA in U87 cells. Very few tissue samples from patients showed weak expression of *env* mRNA, but none of the *rec* or 1.5 kb transcripts. Primary cells expressed the 1.5 kb transcript weakly in early passages, but lost HERV-K expression with extended culture time.

**Conclusions:**

These data suggest that HERV-K splice products do not play a role in human malignant gliomas and therefore, are not suitable as targets for new therapy regimen.

## Findings

### Background

**Figure 1:**
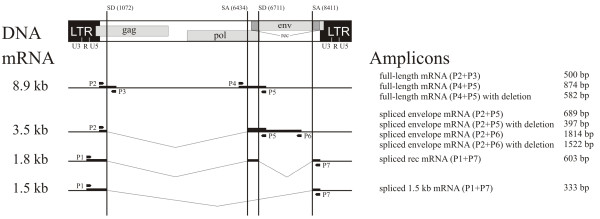
**Organization of HERV-K proviruses, expression pattern and primer localization.** The localization of the primer pairs (P1 to P7), the splice acceptor (SA) and the splice donor (SD), the size of the mRNA in kilo base pairs (kb) and of the corresponding amplicons in base pairs (bp) are indicated. Modified from [10].

Human endogenous retroviruses (HERVs) have been integrated into the genome of human ancestors millions of years ago after ancient germline infections and passed on to the following generations. Today these HERVs comprise about 8% of the human genome [1]. More than 22 HERV families have been identified [2]. They are classified according to the tRNA primer-binding site which is, for example, in the HERV-K family the lysine (K) tRNA [3,4]. Most of the HERV sequences are silenced due to mutations, deletions or other genetic changes. However, HERV-K is transcriptionally active and able to encode all elements necessary for a functional retrovirus [5-8]. Nevertheless, to date no infectious HERV-K has been described [9,10]. The HERV-K genome consists of three genes for functional and structural proteins which are flanked at the 5′ and the 3′ ends by two long terminal repeat (LTR) sequences (Figure 1). The LTR regions contain regulatory elements like enhancer, promoters, polyadenylation signals and recognition sites for regulatory proteins [11]. The three main genes of HERVs are *gag*, *env* and *pol. Gag* encodes structural genes for the formation of the viral matrix like the capsid structure. The two enzymes reverse transcriptase and integrase are encoded by the gene *pol*. The former is necessary for the reverse transcription of the viral RNA sequence into DNA and the latter for the integration of this DNA into the genome of the cell. Finally, *env* encodes for envelope proteins involved in receptor recognition and membrane fusion, as well as the accessory protein Rec [3,5,12]. The latter is a regulatory factor localized in the nucleus of cells. It has been shown that expression of Rec supports cell transformation and induces tumor formation in nude mice [13-15]. Another splice product of the *env* gene is a 1.5 kb transcript with unknown function. Alongside the HERV-K prototype structure, a second type of HERV-K proviruses exists. This type is characterized by a fusion of the *pol* and the *env* genes due to a 292 bp deletion and the loss of the *rec* gene [9]. In addition, an open reading frame for the protein Np9 exists. Like Rec, this protein is found in the nucleus, and discussed to have oncogenic potential [15-17].

Transcripts of HERVs have been detected in a multitude of human cancers, but not in the corresponding normal tissue. These findings have been reviewed comprehensively [2,18]: breast cancer, ovarian cancer, lymphoma, melanoma, germ line tumors, haematological neoplasms, sarcoma, bladder cancer, prostate cancer, primary skin tumors and lymphatic metastasis are examples for putative associations between HERV protein expression and cancer development [2,18]. Moreover, in blood sera of patients suffering from melanoma and ovarian cancers even antibodies directed against HERV-K *gag* and *env* transcripts are detectable [10,19-21]. It has been discussed that these antigens on the surface of cancer cells may be potential targets for a cancer immune therapy [2,22,23]. Indeed, antibodies directed against the HERV-K envelope antigen blocked proliferation of breast cancer cells *in vitro* and inhibited tumor growth in mouse xenograft models [24].

Patients with glioblastoma multiforme (GBM), the most common malignant brain tumor in adults [25], have a very limited prognosis due to the aggressive local and infiltrative growth pattern of the tumor [26,27]. Standard therapy comprises neurosurgical tumor resection followed by irradiation and concomitant temozolomide (TMZ) chemotherapy and adjuvant TMZ-treatment. Although the overall outcome of GBM patients has improved with the introduction of TMZ, the median survival time still does not exceed 14.6 months after first diagnosis [28]. To overcome the limitations of current treatment regimen, new therapeutic targets have to be defined for tumor cell-detection and eradication. Immunotherapy directed against tumor specific antigens may be an option and HERV-K splice products could serve as target. Furthermore, HERV-K derived proteins may play a role during the tumorigenic processes. However, to the best of our knowledge, there are no reports addressing the expression and potential function of HERV-K in human malignant gliomas. Therefore, we elucidated whether HERV-K may be expressed and might play a tumorigenic role in these malignancies.

### Methods

*Tissue samples, cell lines and cell culture.* Expression of HERV-K was analyzed using normal brain and astrocytic tumor specimens. Informed consent of the patient was obtained for the acquisition of tumor material as approved by the ethics committee of the University of Würzburg (medical faculty). The samples used have been discussed in detail in a previous publication [29]. In addition, five human GBM cell lines (U87, U138, U251, U343 and GaMG) were investigated. The teratoma cell line PA1 served as positive control. These cell lines have been purchased from CLS (Cell Lines Service, Eppelheim, Germany). Primary cells were isolated from patients’ GBM tissue samples as described previously [30]. All GBM cells were grown as reported [31] in 75 cm flasks (Corning, New York, USA) at 37°C in an atmosphere of 5.0% CO_2_ and 100% humidity. The PA1 cell line was grown in RPMI-1640 medium supplemented with 10% fetal calf serum and 25 μg/ml gentamycin (both from Invitrogen, Carlsbad, USA).

*RNA extraction, cDNA synthesis and semiquantitative RT-PCR.* Trypsinised cells were washed twice with phosphate-buffered saline. Total mRNA was purified from the cell pellet and from 30 mg of tissue samples by the SV Total RNA Isolation System (Promega, Mannheim, Germany) following the manufacturer’s instructions. These instructions recommend a DNAse digest to remove contaminating genomic DNA. 100 μl DNAse mix (80 μl yellow core buffer, 10 μl 0.09 M MnCl_2_, 10 μl DNAse I, all provided in the kit) were added to each column. The digest was performed for 30–60 min at 37°C. Then the purification was continued according to the manual. Purified RNA was eluted from the columns with 100 μl RNase-free water and samples were stored at -80°C. As a test for contamination with genomic DNA, which would interfere with full legth HERV-K mRNA detection, a PCR reaction was performed using an aliquot of the mRNA before reverse transcription and the PCR-conditions described below. In case of positivity the DNAse digest was repeated as described above for 60 min at 37°C.

One to 5 μg of the total RNA was reverse-transcribed using the RevertAid H Minus First Strand cDNA Synthesis Kit (Fermentas, Ontario, Canada) and the provided oligo(dT)18 primer as described elsewhere [29].

The HERV-K expression level was determined by semiquantitative RT-PCR. The amount of cDNA was normalized to the intensity of the PCR product of the ubiquitously expressed gene glyceraldehyde-3-phosphate dehydrogenase (GAPDH), which was used as internal control [32,33]. The primer sequences for GAPDH detection were sense 5′-GCAGGGGGGAGCCAAAAGGG-3′ and antisense 5′-TGCCAGCCCCAGCGTCAAAG-3′. The full-length HERV-K virus and different splice variants were analysed using a set of seven primers corresponding to HERV-K108/-K10, -K.HOM and related HERV-K (Figure 1). Details about primer sequences, location of primer recognition sites within the virus genome and amplified fragment sizes can be found in [10]. Primer combinations P2 + P3, as well as P4 + P5 confirm the expression of the full length mRNA, while P4 + P5 also distinguish between the prototype and the deletion mutant. Primer combinations P2 + P5 and P2 + P6 detect the envelope sequence and P1 + P7 amplify the double spliced rec and the 1.5 kb mRNA. The polymerase chain reaction was performed using 0.625 U Dream Taq polymerase (Fermentas, Ontario, Canada) in each 25 μl reaction, containing 10x buffer with 25 mM MgCl_2_ (Fermentas, Ontario, Canada). For primer combination P1 + P7 1 μl formamid (Eurobio, Courtaboef Cedex, France) was added to the reaction. Thermocycle parameters were as follows: 10 min at 94°C; 35 cycles (21 for GAPDH) of 30 sec at 94°C, 30 sec at 60°C (68°C for GAPDH), 2 min (1 min for GAPDH) at 72°C; and 10 min at 72°C. The amplification products were separated on 1% agarose gels (Sigma-Aldrich, Steinheim, Germany) containing 0.07 μg/ml ethidium bromide (Roth, Karlsruhe, Germany) and photographed using the BioDocAnalyze digital (Biometra, Göttingen, Germany).

### Results and conclusion

**Figure 2:**
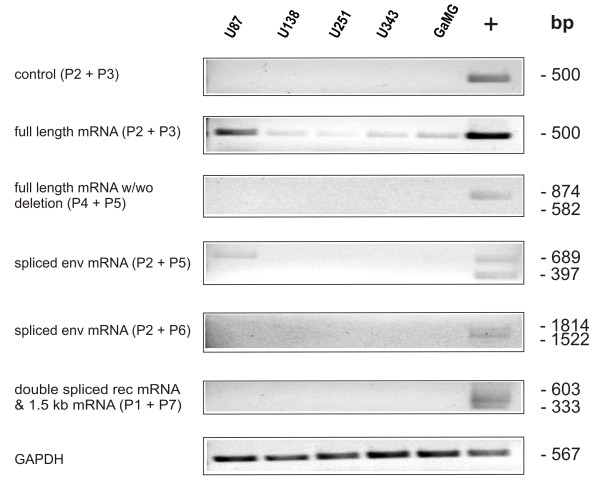
**Expression analysis of five human GBM cell lines by semiquantitative RT-PCR.** Total RNA from the five GBM cell lines U87, U138, U251, U343 and GaMG was used for semiquantitative RT-PCR analysis. The primers for detection of the full length HERV-K mRNA, the deletion mutant, the spliced envelope (*env*) mRNA, the double spliced *rec* and the 1.5 kb mRNA are described in [10]. Their localization within the HERV-K provirus is shown in Figure 1. The length of the cDNA amplicons in base pairs (bp) is shown on the right side of the figure. As positive control (+) cDNA of the teratoma cell line PA1 was used. The various cDNA concentrations were normalized to that of the housekeeping gene GAPDH. As a test for contamination with genomic DNA, which would interfere with full length HERV-K mRNA detection, a PCR reaction was performed using an aliquot of the mRNA before reverse transcription (Control).

Expression of full length HERV-K mRNA and its splice variants *env*, *rec* and 1.5 kb mRNA were analyzed by semiquantitative RT-PCR in different human GBM cell lines, patients’ tissue samples of astrocytic tumors and primary cell cultures of different passages. The human GBM cell lines U138, U251, U343 and GaMG displayed weak expression of the full length HERV-K mRNA (Figure 2), while U87 cells showed strong expression. While most of these GBM cell lines have been tested for the first time, the U87 data confirm already published results [10]. The detection of full length mRNA was not due to contamination of the reaction with genomic DNA (Figure 2). However, the deletion mutant could not be detected in these cell lines. U87 cells showed a very weak expression of the 689 bp envelope mRNA. All other splice variants were not detectable in all GBM cell lines tested (Figure 2).

**Figure 3:**
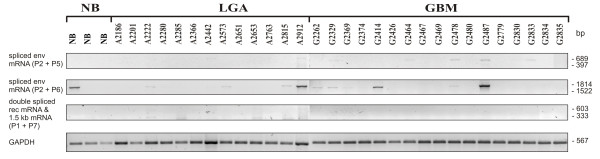
**Expression analysis of HERV-K mRNA splice products in normal brain and human astrocytic tumor samples.** Semiquantitative RT-PCR was used to detect spliced *env* mRNA, double spliced *rec* mRNA and 1.5 kb mRNA of HERV-K in three normal brain samples (NB), 13 astrocytoma WHO grade 2 (low grade astrocytoma, LGA) and 17 glioblastoma multiforme (GBM), as described in Figure 2. GAPDH served as internal loading control for normalization of the cDNA concentrations.

In patients’ tissue HERV-K expression was tested in a standardized cDNA panel of three normal brain (NB) tissue samples, 13 astrocytomas WHO grade 2 (low grade astrocytoma, LGA) and 17 GBMs (Figure 3). Due to the limited availability of patients’ tissue, only the oncogenically relevant splice variants were checked. In one NB (33%), one LGA (7.7%) and two GBM samples (11.8%) expression of the *env* mRNA could be detected. However, the splice products *rec* and 1.5 kb mRNA were not found (Figure 3). This suggests that HERV-K splice products do not play a role in human glioma tumorigenic processes and therefore are not suitable as a therapeutic target.

**Figure 4:**
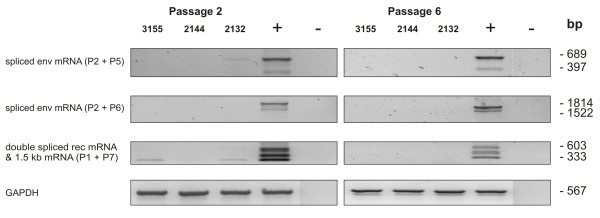
***Expression analysis of HERV-K mRNA splice products in primary GBM cells by semiquantitative RT-PCR.*** Three different primary cell cultures, derived from patients’ biopsies, were passaged 6 times. Total RNA was isolated from passage 2 and passage 6 and used for semiquantitative RT-PCR as described in Figure 2.

Since passaging of cells *in vitro* is able to induce mRNA and protein expression of certain proteins [34-36], we analysed a possible induction of HERV-K expression by cell-passaging. Three primary cell cultures were cultured for 6 passages and HERV-K expression was analysed at passage 2 and passage 6 by semiquantitative RT-PCR (Figure 4). Although at passage 2 the 1.5 kb mRNA was expressed very weakly by all three cultures, there was no expression of any splice product detectable at passage 6 (Figure 4). This may be due to the culture conditions, which seem to adjust the HERV-K expression pattern of primary cells to that seen in established GBM cell lines.

In summary, we found HERV-K full length mRNA expressed in human GBM cell lines. However, the splice variants were neither detectable in GBM cell lines, nor in most of the patients’ tissue samples from NB, LGA and GBM. Passaging of primary cells derived from these tumors did not induce HERV-K expression.

Therefore, we conclude that HERV-K, at least the variants which can be detected with the primer pairs we used, is - in contrast to several other cancer types [2] - not expressed in human malignant gliomas, and therefore probably does not play a role in proliferation and progression of these tumors. Our findings do not exclude that other HERVs or other virus types may be involved in modulating the phenotype of GBM cells. For example, it has been shown that sequences and viral gene expression of the human cytomegalovirus (HCMV) can be found in GBM and may act tumor supportive by interaction with key signaling pathways [37].

## Abbreviations

Bp: Base pairs; cDNA: Copy DNA; DNA: Deoxyribonucleic acid; GAPDH: Glyceraldehyd-3-phosphate dehydrogenase; GBM: Glioblastoma multiforme; HCMV: Human cytomegalovirus; HERV-K: Human endogenous retrovirus K; K: Lysine; kb: Kilo base pairs; LGA: Astrocytoma WHO grade 2; LTR: Long terminal repeat; mRNA: Messenger ribonucleic acid; NB: Normal brain; RT-PCR: Reverse transcription polymerase chain reaction; TMZ: Temozolomide.

## Competing interests

The authors declare that they have no competing interests.

## Authors’ contributions

CH, UK and JD participated in the design of the study. AFK, MW and CH carried out the experimentation procedures and together with JD performed the data analysis and interpretation. CH, GHV and ML coordinated the work. AFK and CH drafted the manuscript, with help and critical revision by UK, GHV, TL and R-IE. All authors read and approved the final manuscript.
